# MYO9A deficiency in motor neurons is associated with reduced neuromuscular agrin secretion

**DOI:** 10.1093/hmg/ddy054

**Published:** 2018-02-16

**Authors:** Emily O’Connor, Vietxuan Phan, Isabell Cordts, George Cairns, Stefan Hettwer, Daniel Cox, Hanns Lochmüller, Andreas Roos

**Affiliations:** 1 John Walton Muscular Dystrophy Research Centre, Institute of Genetic Medicine, Newcastle University, Newcastle upon Tyne, UK; 2 Leibniz-Institut für Analytische Wissenschaften-ISAS e.V, Dortmund, Germany; 3 Wellcome Trust Centre for Mitochondrial Research, Institute of Genetic Medicine, Newcastle University, Newcastle upon Tyne, UK; 4 Neurotune AG, 8952 Schlieren, Switzerland

## Abstract

Congenital myasthenic syndromes (CMS) are a group of rare, inherited disorders characterized by compromised function of the neuromuscular junction, manifesting with fatigable muscle weakness. Mutations in *MYO9A* were previously identified as causative for CMS but the precise pathomechanism remained to be characterized. On the basis of the role of MYO9A as an actin-based molecular motor and as a negative regulator of RhoA, we hypothesized that loss of MYO9A may affect the neuronal cytoskeleton, leading to impaired intracellular transport. To investigate this, we used MYO9A-depleted NSC-34 cells (mouse motor neuron-derived cells), revealing altered expression of a number of cytoskeletal proteins important for neuron structure and intracellular transport. On the basis of these findings, the effect on protein transport was determined using a vesicular recycling assay which revealed impaired recycling of a neuronal growth factor receptor. In addition, an unbiased approach utilizing proteomic profiling of the secretome revealed a key role for defective intracellular transport affecting proper protein secretion in the pathophysiology of MYO9A-related CMS. This also led to the identification of agrin as being affected by the defective transport. Zebrafish with reduced MYO9A orthologue expression were treated with an artificial agrin compound, ameliorating defects in neurite extension and improving motility. In summary, loss of MYO9A affects the neuronal cytoskeleton and leads to impaired transport of proteins, including agrin, which may provide a new and unexpected treatment option.

## Introduction

The neuromuscular junction (NMJ) is a tightly controlled functional unit, with highly specialized pre- and post-synaptic regions that must function in a coordinated manner for effective NMJ transmission to be achieved. The complex organization of the neuronal cytoskeleton is crucial for both NMJ formation and functionality. Actin in particular is a highly important component of the cytoskeleton as it enables complex and dynamic movement of cargo for junctional signalling and formation by using members of the myosin superfamily. Rho-GTPases and Rho-GEFs (GDP/GTP nucleotide exchange factors) are critical in the control of actin dynamics and disturbed Rho has already been implicated in the vulnerability of the peripheral nervous system (1,2). Other cytoskeletal components, also important for NMJ functionality, include microtubules which facilitate the long-distance transport necessary for motor neurons and neurofilaments that provide pre-dominantly structural support to neurons but are implicated in a range of peripheral neuropathies (3–5).

Various mutations in critical NMJ proteins are known to cause primary defects in neuromuscular transmission and lead to the clinical picture of congenital myasthenic syndromes (CMS). The main symptom of patients with CMS is fatigable muscle weakness that usually starts in childhood and can disrupt the skeletal, respiratory, bulbar and ocular muscles depending on the protein involved. CMS constitute a group of genetically heterogenic disorders and causative genes can be broadly categorized as pre-synaptic, synaptic or post-synaptic. Recently, we expanded the catalogue of known pre-synaptic CMS causative genes by describing recessive missense mutations in the unconventional myosin encoding gene, *MYO9A*, present in three patients showing a severe neonatal phenotype with both respiratory and bulbar involvement (6).

Unconventional myosins are actin based molecular motors that are present in almost all eukaryotic cells. There are two class 9 myosin proteins expressed in humans; MYO9A and MYO9B. MYO9A follows the same basic structural composition as other unconventional myosins, containing a force generating myosin motor domain in the head region which binds actin, as well as an extended tail region that contains a Rho-GAP (Rho GTPase-activating protein) domain. The presence of this Rho-GAP domain, unique to class 9 myosins, infers the ability to negatively regulate the activity of Rho by converting it from its active GTP-bound state to an inactive GDP-bound state, with the pathway summarized in Supplementary Material, Figure S1A (7). Because of direct interaction with actin and influence of actin via the Rho-GAP domain, MYO9A has been implicated in the regulation of filamentous-actin (f-actin) dynamics, a process that is crucial for the structure of the pre-synaptic nerve terminal, vesicular trafficking and neurotransmitter release, as highlighted in Supplementary Material, Figure S1B (8–11).

Following preliminary investigations, we were already able to demonstrate an *in vitro* role for MYO9A in neurite branching and extension utilizing the mouse motor neuron-like hybrid cell-line (NSC-34). Depletion of the MYO9A orthologues from zebrafish, myo9aa and myo9ab, also supported a role for this unconventional myosin in formation of the NMJ and in movement of the developing zebrafish. Neurons are extremely vulnerable to transport deficiencies and thus any defects here may clearly affect the finely balanced organization of the NMJ. Defects in plectin, a cross-linking protein for intermediate filaments, have already been associated with CMS (12). However, the precise molecular mechanisms in CMS caused by perturbed cytoskeleton still remain elusive. Therefore, in this study we aim to systematically widen the pathological implications of cytoskeletal involvement in CMS. On the basis of our previous results and other identified cytoskeletal and exocytotic functions of MYO9A, here our hypothesis was that MYO9A disrupts NMJ function in CMS by affecting the neuronal cytoskeleton, thus impacting on vesicular trafficking and protein secretion. To address this hypothesis, we have employed both biased and unbiased approaches: immunological based assays to assess structural integrity and vesicular trafficking proficiency of NSC-34 cells depleted for MYO9A and unbiased proteomic profiling of the secretome of control and MYO9A-depleted NSC-34 cells.

## Results

### MYO9A-depletion affects the cytoskeleton of NSC-34 cells

**Figure 1.**
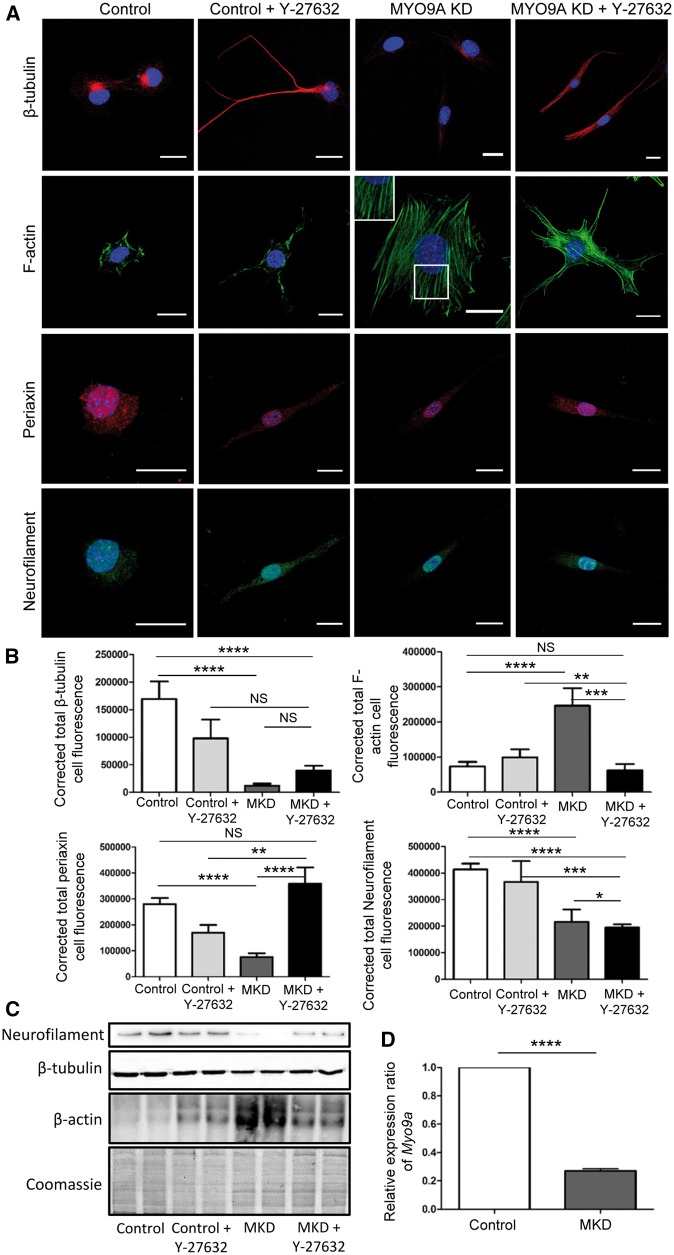
Effect of MYO9A-depletion on the cytoskeleton. (**A**) Immunofluorescent staining of control and MYO9A-depleted NSC-34 cells with and without over-night treatment of Y-27632. Cytoskeletal components β-tubulin, periaxin, neurofilament and actin, were assessed. Longer neurites are observed in treated cells, and MYO9A-depleted cells display increased stress fibre-like structures as shown in the inset image. Scale bars = 20µm. (**B**) Graphs showing the corrected total cell fluorescence for levels of β-tubulin (*n* > 26), periaxin (*n* > 30), neurofilament (*n* > 87) and actin (*n* > 33) in each cell/treatment condition. Mann–Whitney test, NS = not significant, *P ≤ 0.05, **P ≤ 0.01, ***P ≤ 0.001, ****P ≤ 0.0001, MKD = MYO9A-depleted, error bars = mean + standard error of the mean. (**C**) Immunoblot analysis for actin (42 kDa), β-tubulin (50 kDa) and neurofilament (light chain—70 kDa) with a Coomassie loading control, in control and MYO9A-depleted cells. MKD = MYO9A-depleted. (**D**) Graph demonstrating the relative expression ratio of *Myo9a* in control and *Myo9a*-shRNA transfected NSC-34 cells as obtained from qPCR results, utilizing *Pgk1* as a control. MKD = MYO9A-depleted, error bar represents mean + standard error of the mean, unpaired *t*-test **P < 0.01.

In order to observe the cytoskeleton in MYO9A-depleted NSC-34 cells, immunofluorescent staining and immunoblot analysis of F-actin, β-tubulin, periaxin and neurofilament was performed. This revealed an increase in fluorescence intensity of f-actin in the MYO9A-depleted NSC-34 cells (Mann–Whitney test, *P* = 0.0016, *n* > 34, Fig. 1A and B), confirmed by western blot as shown in Figure 1C. There is also an increase in the number of cells displaying actin stress fibre-like structures in MYO9A-depleted cells as compared with controls, from 5% to 85% respectively (*n* > 65, Supplementary Material, Fig. S2A). This is further supported by orientation analysis of actin positive fibres, revealing a significant decrease in the dispersion (an increase in organization) of fibre direction in MYO9A-depleted cells (Mann–Whitney test, *P* < 0.0001, *n* > 65, Supplementary Material, Fig. S2B). Neurofilament, periaxin and β-tubulin, important structural components of neuronal cells, all had decreased fluorescence intensity (Mann–Whitney test, *P* < 0.0001, *n* > 28) in cells depleted for MYO9A (Fig. 1A and B), with β-tubulin and neurofilament (light chain) also confirmed via western blot (Fig. 1C).

As MYO9A has been shown to act as an inhibitor of RhoA activity, cells were also treated with a compound to inhibit the downstream function of the RhoA pathway (ROCK inhibitor, Y-27632). The contribution of the RhoA pathway to the cytoskeletal defects observed could then be determined (RhoA pathway outlined in Supplementary Material, Fig. S1A). Actin levels were increased in control cells following application of Y-27632, and decreased in MYO9A-depleted cells to a level similar to untreated controls, as shown in Figure 1A, B and C (Mann–Whitney test, *P* = 0.484, *n* > 25). β-Tubulin also returned to a level not significantly different to that of treated control cells (Mann–Whitney test, *P* = 0.126, *n* > 26), however, this remained significantly less than the untreated controls (Mann–Whitney test, *P* < 0.0001, Fig. 1A, B and C). Y-27632 also increased the level of periaxin fluorescence in the MYO9A-depleted cells to a level comparable to untreated controls (Mann–Whitney test, *P* = 0.912, *n* > 30, Fig. 1A and B). Inhibition of the RhoA pathway was unable to rescue the decrease in neurofilament, furthering the significant decrease in levels as compared with untreated controls (Mann–Whitney test, *P* < 0.0001, *n* > 87, Fig. 1A, B and C). Therefore, a proportion of the effect of MYO9A-depletion on the cellular cytoskeleton can be attributed to interaction with the RhoA pathway. Treatment with Y-27632 also induced neurite extension in both control and MYO9A-depleted NSC-34 cells, suggesting that the already described neurite extension in NSC-34 cells depleted for MYO9A results from perturbed RhoA signalling.

As described above, we discovered a dysregulation of actin in MYO9A-depleted NSC-34 cells. Actin was previously used to confirm the depletion of MYO9A (6). Therefore, we performed a qPCR to confirm MYO9A knockdown using an alternative control gene, *Pgk1*, to normalize results between control and MYO9A-depleted cells. MYO9A-depleted cells display a 4-fold significant reduction in *Myo9a* expression as compared with the control NSC-34 expression levels (unpaired *t*-test, *P* = 0.0022, Fig. 1D).

### Vesicular recycling is impaired in NSC-34 cells depleted for MYO9A

Lack of MYO9A is hypothesized to impact vesicular recycling processes, including endosomal recycling in nerve cells, due to perturbed cytoskeleton. To assess this, a paradigmatic surface receptor, TrkA, fused with a GFP-tag was transiently transfected into NSC-34 control and MYO9A-depleted cells. Following application of NGF, a proportion of the receptors will be internalized and their return to the surface can be tracked over-time.

**Figure 2.**
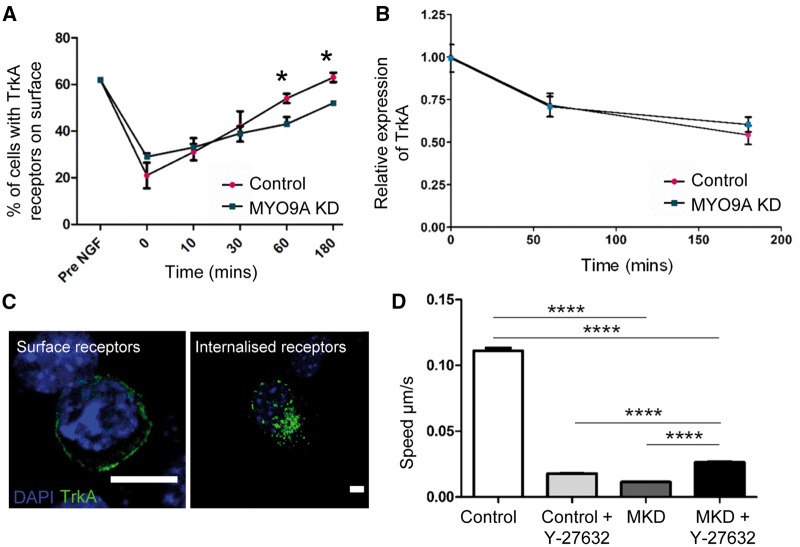
Effect of MYO9A on vesicular recycling. (**A**) Graph depicting the change in TrkA receptor localization over-time in control and MYO9A KD cells following NGF application to induce internalization (Mann–Whitney test, *P ≤ 0.05). Values have been normalized to pre-NGF levels to allow direct comparison at each time point and error bars represent the mean ± standard error of the mean, NGF = nerve growth factor. (**B**) Expression of TrkA at time 0, 60 and 180 min, following application of nerve growth factor. Results shown are relative to control cells at time 0 min. Error bars represent mean ± standard error of the mean. (**C**) Representative images of internalized and surface TrkA-GFP receptors to show how receptors were classified, scale bars = 20µm. (**D**) Live-cell time lapse microscopy allowed speed of internalized, GFP positive TrkA receptors to be quantified over a time period of 10 min for cells untreated or treated with a ROCK inhibitor (Y-27632). Absolute values are shown and error bars represent mean + standard error of the mean, Mann–Whitney test ****P ≤ 0.0001. MKD = MYO9A-depleted.

Results obtained from both control and MYO9A-depleted cells were normalized using pre-NGF values to allow direct comparison at each time point. Following NGF application, the NSC-34 cells demonstrated endocytosis of the TrkA receptors and by 60 min almost all control cells had successfully exocytosed the receptors to pre-NGF levels, which was complete by 180 min (Fig. 2A). In the MYO9A-depleted cells, by 60 min significantly less cells had managed to return receptors to the surface as compared with control NSC-34 cells (Mann–Whitney test, *P* = 0.029, *n* = 200). By 180 min 10% fewer cells were expressing TrkA receptors on the surface than before NGF application, and this was significantly less than the control NSC-34 cells (Mann–Whitney test, *P* = 0.028, *n* = 200). Representative images showing internalized and externalized receptors are shown in Figure 2C. To determine whether the lack of receptor expression on the surface of MYO9A-depleted NSC-34 cells at 180 min was due to more receptors being targeted for degradation rather than recycling, we also performed immunoblots on the cells at time points 0, 60 and 180 min. This revealed that there was no increased degradation of TrkA receptors in MYO9A-depleted cells as compared with the controls (Mann–Whitney test, *P* = 0.400) and thus the lack of exocytosis is most likely based on a defect, in part, of the recycling pathway (Fig. 2B).

Time-lapse microscopy, in which the TrkA-positive vesicles were tracked over a period of ten minutes, revealed a significant decrease in the speed of the receptor trafficking in MYO9A-depleted cells as shown in Figure 2D (Mann–Whitney test, *P* < 0.0001, *n* = 10). An example time-lapse video is shown in Supplementary Material, Video S1, as well as evidence that global endocytosis and exocytosis is impaired through the use of an FM 1–43 dye (Supplementary Material, Fig. S3). Following on from our results using the ROCK inhibitor to partially rescue the cytoskeletal defects, we also treated cells with Y-27632 and performed the time-lapse analysis. A significant increase in speed of GFP-positive vesicles in MYO9A-depleted cells treated with Y-27632 was achieved as compared with untreated MYO9A KD cells, as well as treated control cells (Mann–Whitney test, *P* < 0.0001, *n* = 12). Hence, our combined findings clearly suggest an impaired vesicular transport upon MYO9A-depletion *in vitro*, partially due to perturbed cytoskeletal organization as a pre-requisite of proper transport of proteins packed in vesicles.

### Secretomic analysis of NSC-34 cells

**Figure 3.**
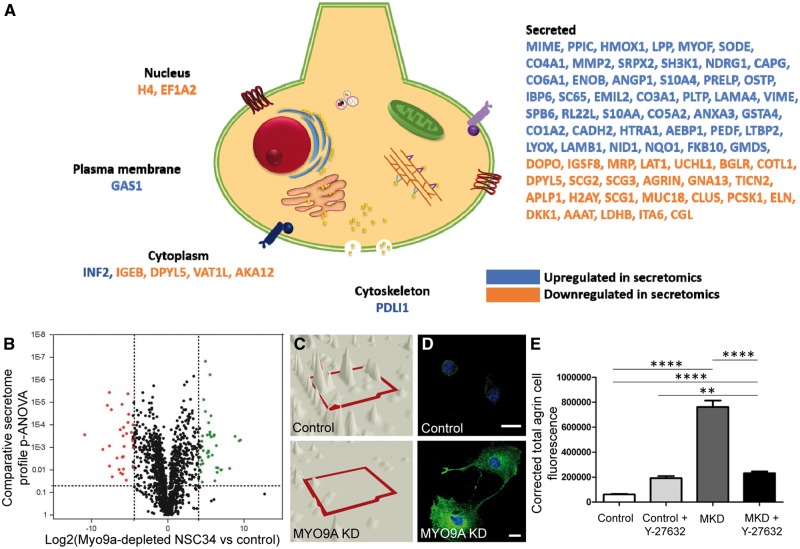
Secretomic analysis of control and MYO9A-depleted NSC-34 cells. (**A**) Schematic cell diagram outlining in which cellular compartments the dysregulated proteins are implicated. (**B**) Volcano plot for secretomic analysis of control NSC-34 cells compared with MYO9A KD cells. (**C**) 3D montage of agrin peptide peaks (LYVGGLPEEQVATVLDR) as identified in the secretomic profile in control and MYO9A KD NSC-34 cells. An Absence of agrin can be observed in the MYO9A KD cells. (**D**) Immunofluorescent staining of control and MYO9A-depleted NSC-34 cells showing an upregulation of agrin within the cell (corroborating with reduced secretion), scale bars = 20µm. (**E**) Graph showing the corrected total cell fluorescence for levels agrin in each cell/treatment condition (*n* > 92). Mann–Whitney test, **P ≤ 0.01, ****P ≤ 0.0001, MKD = MYO9A-depleted, error bars = mean + standard error of the mean.

Following the observation that intracellular transport is impaired in cells depleted for MYO9A, a secretome study was performed in order to determine whether this impairment also affected proper secretion of proteins from the nerve cells. Proteins secreted from NSC-34 wild-type and MYO9A-depleted cells were collected and subjected to label-free shotgun proteomic analysis. This approach captured the abundance of 1532 proteins and of these we found there to be 46 proteins with increased and 30 with decreased extracellular abundance, highlighting the defect we are seeing in impaired protein transport and release (Fig. 3A and B, Supplementary Material, Table S2). Of particular importance is the identification of a 5.4-fold decrease in secretion of agrin from the MYO9A-depleted NSC-34 cells, a key mediator in the development of the NMJ and a well characterized protein involved in CMS pathology (13–15). Reduced agrin secretion is highlighted in Figure 3C, with an absence of ion peaks corresponding to the LYVGGLPEEQVATVLDR peptide in a 3D montage unique to agrin as detected in the secretomic analysis. In this context, a significant increase in the intracellular immunofluorescent signal for agrin is also demonstrated in cells lacking MYO9A, in Figure 3D and E, to further support this finding (Mann–Whitney test, *P* < 0.0001, *n* > 92). Corroborating with our hypothesis that transport/secretory defects can be partly attributed to the dysregulation of the RhoA pathway, application of Y-27632 was able to significantly reduce the intracellular accumulation of agrin, however, this was still significantly higher than treated control cells (Mann–Whitney test, *P* = 0.003). Other interesting proteins identified in the secretomic analysis include neuronal ubiquitin hydrolase (UCHL1), inverted formin 2 (INF2), dihydropyrimidinase-related protein 5 (DPYL5) and peptidyl-prolyl *cis*-*trans* isomerase FKBP10 (FKBP10).

Analysis of sub-cellular localizations of the proteins identified with altered abundances has been performed utilizing Uniprot (http://www.uniprot.org/; date last accessed November 5, 2017), Pubmed (https://www.ncbi.nlm.nih.gov/pubmed/; date last accessed November 5, 2017), LOCATE (sub-cellular localization database: http://locate.imb.uq.edu.au/; date last accessed November 5, 2017) and MGI (Mouse Genome Informatics: http://www.informatics.jax.org/; date last accessed November 5, 2017) and revealed known secretion or extracellular localization of 64 out of the affected 76 proteins (Supplementary Material, Table S2).

The mass spectrometry proteomics data have been deposited to the ProteomeXchange Consortium via the PRIDE partner repository with the dataset identifier PXD007178.

### Treatment of zebrafish with agrin compound—NT-1654

Neural agrin, a protein identified with decreased abundance in secretome data for MYO9A-depleted cells, is an important mediator of NMJ development and maintenance and mutations in *AGRN* itself can cause CMS (13). Therefore, we hypothesized that the lack of agrin secretion in MYO9A CMS may be contributing to the phenotype we observe and thus we tested the effect of treating the *myo9aa/ab* MO zebrafish with a novel agrin compound; NT-1654. This compound is composed of a 44 kDa fragment of agrin that is not only soluble but also resistant to breakdown by neurotrypsin at the NMJ and has already proven successful in the treatment of various mouse models of disease (16). Varying concentrations of NT-1654 were trialled, from 0.15 ng to 2.4 ng per injection. A dosage of 0.15 ng was selected for use as over 50% of the zebrafish survived and we still observed a functional effect. In order to determine the effect of NT-1654 on NMJ development in zebrafish we injected one-cell stage embryos with the compound, or with *myo9aa/ab* MOs (2 ng/0.2 ng respectively), as described (6) or with both the agrin compound and the MOs. Various functional and morphological parameters were then assessed for changes.

**Figure 4.**
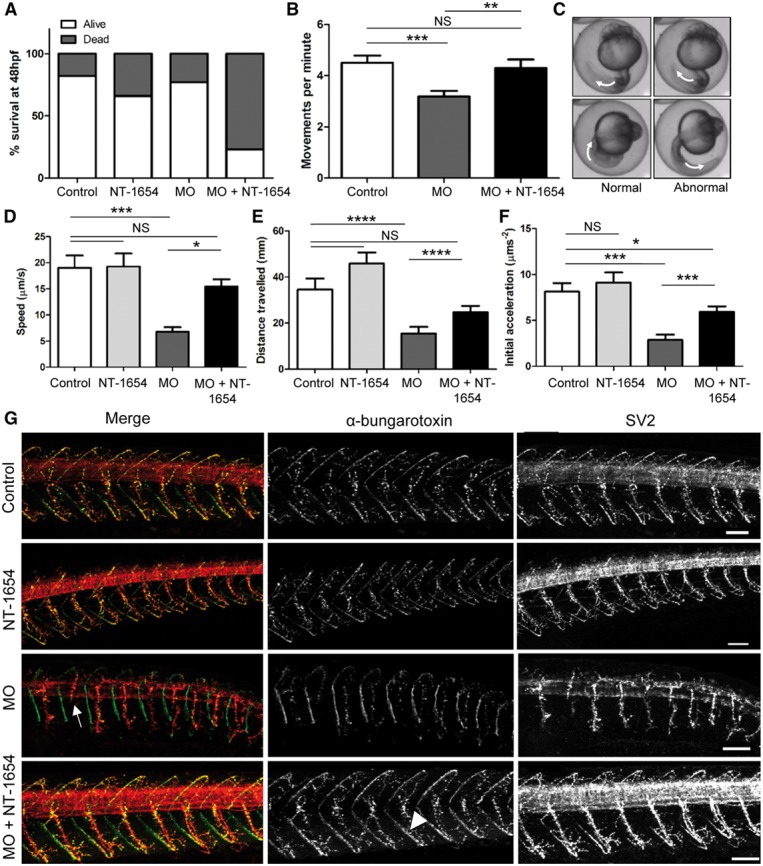
Effect of NT-1654 treatment in MYO9A-depleted zebrafish. (**A**) Graph depicting the survival rate of control, NT-1654 injected, MO injected and MO + NT-1654 injected zebrafish at 48 hpf (*n* = 100). (**B**) Movements performed by zebrafish within the chorion at 24 hpf in 1 min. Unpaired *t*-test, NS = not significant, **P ≤ 0.01, *n* > 40. (**C**) Example of chorion movement showing a full rotation of the zebrafish tail indicated by white arrows. (**D**) Speed, (**E**) distance and (**F**) initial acceleration of zebrafish swimming away following tactile stimulation at 48 hpf as calculated using TrackMate (ImageJ), *n* > 34. Unpaired *t*-test, NS = not significant, *P ≤ 0.05, **P ≤ 0.01, ***P ≤ 0.001, ****P ≤ 0.0001, error bars represent mean + standard error of the mean. (**G**) Immunofluorescent staining of 48 hpf zebrafish (control, NT-1654/MO/MO + NT-1654 injected) to visualize the neuromuscular junctions. SV2 stains pre-synaptic motor neurons and α-bungarotoxin detects the post-synaptic AChRs. MO injected fish display shortened axons (white arrow) and this is rescued by the application of NT-1654. AChR clusters also appear more prominent following addition of NT-1654 (white arrow head). Scale bars = 50µm. MO = morpholino, hpf = hours post-fertilization, AChRs = acetylcholine receptors, SV2 = synaptic vesicle protein 2.

Injection of the agrin compound alone lead to 26% more embryos dying by 48 hpf, whereas the MO decreased survival rate by 5% and both combined by 59% as compared with control zebrafish (*n* = 100, Fig. 4A). However, as this study has been designed to be a proof of principal, demonstrating the beneficial effects of agrin restoration *in vivo*, the dose was not further optimized.

Spontaneous movement of zebrafish within the chorion, characterized by repetitive tail movements, begins at 17 hpf, peaks at 19 hpf and continues to 27 hpf. These movements are thought to be indicative of neuronal development and are independent of supraspinal input (17,18). Control zebrafish moved on average 4.5 times in 1 min, while MO injection reduced this to 3.186 (unpaired *t*-test, *P* = 0.0009) and a combined treatment of MO with NT-1654 was able to rescue this to 4.295 (unpaired *t*-test versus control *P* = 0.6435, versus MO *P* = 0.0079, *n* > 40), as shown in Figure 4B. A representative image of the spontaneous movements recorded in the fish is depicted in Figure 4C.

CMS patients exhibit varying degrees of muscle weakness that is fatigue related, and therefore we assessed the ability of the zebrafish to swim while measuring various outputs. At 27 hpf some zebrafish begin to display signs of swimming behaviour, defined as small tail movements that propel the zebrafish forward by at least one body length, however, these become much more pronounced and easy to measure later in development around 48 hpf (18). Therefore at 48 hpf we performed a touch-evoked tactile stimulation response assay, whereby the zebrafish are touched on the back of the head by a pipette tip and the response recorded, example shown in Supplementary Material, Video S2. A characteristic movement is usually performed in which the zebrafish flips backwards and swims away from the stimuli. Here, we utilized a modified tracking protocol that employs the use of the TrackMate plugin (ImageJ), to allow the assessment of swimming speed, initial zebrafish acceleration and also the distance swam after tactile stimulation (average *n* = 34). Speed of swimming is shown in Figure 4D, and here the addition of NT-1654 is shown to improve velocity from 19.02 µm/s to 19.27 µm/s, although this was not significant (unpaired *t*-test, *P* = 0.9466). *Myo9aa/ab* MO fish exhibited slow swimming responses, which was significantly reduced to 6.786 µm/s (unpaired *t*-test, *P* < 0.0001). NT-1654 treatment rescued this phenotype, improving swimming speed to 15.44 µm/s which did not show significant differences to the controls (unpaired *t*-test, *P* = 0.1675), but was significantly increased as compared with MO-treated larvae (unpaired *t*-test, *P* < 0.0001).

Distance travelled is shown in Figure 4E, and this was also improved by the application of NT-1654, with total displacement increasing between controls and treated fish from 34.58 mm to 45.82 mm respectively, although this increase was not significant (unpaired *t*-test, *P* = 0.1341). *Myo9aa/ab* MO zebrafish were not able to swim as far as controls, averaging a distance of just 15.47 mm (unpaired *t*-test, *P* = 0.006). However, combined treatment of MO with NT-1654 increased the swimming distance after stimulation to 24.67 mm, which was a significant increase compared with *myo9aa/ab* MO zebrafish (unpaired *t*-test, *P* = 0.0244) and was no longer significantly different to controls (unpaired *t*-test, *P* = 0.0622).

Finally, initial acceleration was assessed, which is reported to reach a maximal value within 0.2 s of tactile stimulation and is proportional to the force generating capacity of the skeletal muscle and thus is indicative of proper NMJ function (19). As shown in Figure 4F, acceleration follows a similar pattern to other movement parameters, with control zebrafish on average accelerating by 8.13 mm/s^2^ which is not significantly increased by NT-1654 treatment alone (9.116 mm/s^2^, unpaired *t*-test, *P* = 0.531). In *myo9aa/ab* MO fish acceleration is decreased significantly to 2.865 mm/s^2^ (unpaired *t*-test, *P* < 0.0001), and then increased significantly to 5.918 mm/s^2^ when NT-1654 is applied (unpaired *t*-test, *P* = 0.0007), which is now not significantly different to control zebrafish (unpaired *t*-test, *P* = 0.428).

In order to determine whether these functional improvements seen through application of NT-1654 are also reflected at the level of the NMJ, staining for the pre-synaptic motor neurons and post-synaptic AChRs was performed at 48 hpf (Fig. 4G). The *myo9aa/ab* MO zebrafish exhibited truncated motor axons as described previously (6), and those treated with NT-1654 developed as normal and were often indistinguishable to controls. In zebrafish treated with both the MO and NT-1654, even those with curved tails, demonstrated an improved phenotype. This included restoration of motor neuron branching and extension, reaching normal length as in controls. There was also an increase in AChR cluster intensity as shown in Figure 4G, which could also be observed in wild-type fish treated with NT-1654.

## Discussion

Unveiling the pathophysiology of CMS caused by mutations in the gene encoding unconventional myosin protein, MYO9A, is an important objective for improving our understanding of how defects in cytoskeletal motor proteins might lead to NMJ disorders. On the basis of cytoskeletal and vesicular defects seen in other NMJ/neurodegenerative disorders, including various sub-types of ALS such as ALS2 and other familial forms, as well as CMT2E, we hypothesized that loss of MYO9A might cause a similar effect in CMS and would lead to a disrupted cytoskeleton in our *in vitro* NSC-34 cell model (20–22). There have been previous reports regarding the effects of MYO9A-depletion on the actin filament network in other cell types such as bronchial epithelial cells and kidney tubules, as MYO9A is not only an actin-based molecular motor but also acts as a Rho GTPase (8,10,23). In order to obtain deeper insights into the pathomechanism of MYO9A CMS we therefore performed a combined analysis involving both unbiased proteomics to identify novel disease substrates and a range of functional assays.

Prior to testing our hypotheses, the reduction of MYO9A in our *in vitro* model was re-confirmed using qPCR, as the previously published reference gene for normalization (Actin) was since found to be dysregulated in our cells, and there are no working commercially available anti-bodies for western blot in mice. Therefore, we repeated our qPCR with *Pgk1* as a reference gene, an established normalization control for neuronal cells (24). Immunofluorescent and immunoblotting investigation of NSC-34 cells lacking MYO9A confirmed the presence of defects in the cytoskeleton, including an increase in the expression of actin and a decrease in neurofilament, β-tubulin and periaxin (which interacts with the dystroglycan complex, thus linking the basal lamina to cytoskeleton). An increase in actin was accompanied by the formation of a considerable actin stress fibre network. This corroborates with reports that addition of RhoA to fibroblasts in culture leads to the rapid formation of stress fibres, as we have reduced negative regulation of RhoA with our MYO9A-depletion (25,26). Treatment of these cells with Y-27632, a compound that blocks the action of Rho-associated protein kinase (ROCK, a protein kinase activated by RhoA), was able to rescue the increase in actin and to reduce it to levels similar to control cells. This was also coupled with a decrease in the presence of stress fibres, which is in agreement with the role of ROCK in promotion of stress fibre formation in cells (27). With regards to the other cytoskeletal abnormalities observed, a decrease in neurofilament has also been reported in NSC-34 cells expressing mutant *SOD1*, as an *in vitro* model of the neurodegenerative disease, ALS. However, whether this decrease plays an injurious or protective role remains unclear (28). Treatment of MYO9A-depleted cells with the Y-27632 was unable to rescue the loss of neurofilament observed, suggesting dynamics of neurofilament are not under the control of the RhoA pathway here. β-Tubulin, a component of the microtubule network, was also decreased in MYO9A-depleted NSC-34 cells and this was partly rescued by Y-27632 treatment which has been observed previously in podocyte cells (29). A reduction in complex microtubule network formation has also been observed in other cell models for motor-neuron related diseases, including mutant *SOD1* NSC-34 cells (30). We similarly assessed periaxin using immunofluorescence, as it is a structural protein previously linked to neurodegenerative disorders that affect the peripheral nervous system (31,32). Treatment with Y-27632 was also able to increase periaxin to levels not significantly different from controls, demonstrating that periaxin dynamics are at least partly regulated by the MYO9A-controlled RhoA pathway which has not previously been reported. Hence, these combined findings underline a significant role of MYO9A in cytoskeletal maintenance via modulation of the ROCK pathway.

As disruption of the cytoskeleton was observed, we next wanted to address whether vesicular trafficking and recycling of proteins were functioning appropriately, as both actin and microtubule networks are important for all aspects of vesicular transport, from intracellular movement to regulation of endo and exocytosis (33–35). In order to address this question, we employed the use of a paradigmatic growth factor receptor, TrkA, as internalization can be stimulated by applying NGF. However, the use of TrkA is also relevant as impaired TrkA signalling has been linked to perturbed neurotransmission paralleled by concomitant and progressive loss of selected pre-synaptic and vesicle trafficking proteins, and in relation to CMS, TrkA is also able to modulate the release of ACh from basal forebrain neurons (36,37). A similar assay has been performed by Roos *et al.*, utilizing the epidermal growth factor receptor, and other studies have performed analysis of TrkA-GFP following nerve growth factor application and reported similar internalization of receptors and ability to track movements using live-cell confocal microscopy (38–40). The recycling assay performed here revealed defective recycling in MYO9A-depleted cells, and this was further confirmed by live cell imaging in which slower vesicular trafficking was observed that could be significantly improved by the application of Y-27632, therefore linking the morphological observations of this study with functional changes. There was also no increase in TrkA receptor degradation in MYO9A-depleted cells to account for the lack of receptor surface expression after 3 h. In neurodegenerative diseases such as ALS there is a measurable decrease in the speed of axonal transport in motor neurons, as well as loss of motor proteins that occurs as an early event in the pathophysiology (41), thus defective intracellular dynamics may be a common pathological hallmark. Furthermore, performing an additional experiment, we were able to confirm that there is not only a defect in TrkA dynamics but also a global disruption to endo and exocytosis through the use of an FM 1–43 dye, suggesting that perturbed protein transportation represents a down-stream event of MYO9A pathophysiology.

Prompted by the knowledge that neurotransmitters and other factors important for NMJ function are transported to the active zone of nerve terminals by vesicular-mediated transport before exocytosis into the synaptic cleft, we also hypothesized that secretion might be affected in MYO9A-depleted cells. To test this assumption, we performed secretomic analysis to allow identification of any mis-secreted proteins. Of the 76 proteins with significantly altered abundances, 64 have been reported to be secreted or have an extracellular localization. However, for other proteins found to be increased in the extracellular supernatant of MYO9A-depleted cells, a direct participation in the underlying pathophysiology can be assumed. One of those is INF2, a CMT-associated protein which accelerates polymerization and depolymerization of actin sub-units (42,43). This known function of INF2 directly links to our pathomorphological cytoskeletal findings also affecting actin and suggests that abnormal cytoskeleton upon MYO9A-depletion is caused by more complex molecular processes affecting a variety of proteins responsible for the build-up and maintenance of the structural components. This hypothesis is further supported by the fact that FKBP10 (also increased in the extracellular supernatant of MYO9A-depleted cells), as a molecular chaperone, can regulate muscle actin expression (44). As MYO9A-depleted cells and zebrafish also exhibit defects in axon extension the presence of dysregulated proteins involved in axon extension and guidance are particularly interesting, including DPYL5 that has not previously been reported to be secreted, but also ANGP1, SRPX2 and TICN2 (45–48). While other proteins are known to be secreted, their dysregulation may still be important for MYO9A-related NMJ defects. For example, the neuronal ubiquitin hydrolase, UCHL1, is important for the maintenance and structure of the NMJ and mutations in *UCHL1* can cause a motor neuropathy (49). A knockout UCHL1 mouse exhibits impaired synaptic transmission at the NMJ, as well as loss and accumulation of vesicles at the nerve terminal, thus may be important for the pathology we observe in MYO9A-depleted cells (50). Furthermore, other proteins may suggest the activation of rescue mechanisms, such as the upregulation of pigment epithelium-derived factor (PEDF), a neurotrophic factor that acts as a neuroprotective agent in the nervous system. Finally, one of the most important findings in this secretomic study was the identification of decreased agrin secretion. Agrin is an important mediator of post-synaptic endplate differentiation and function and is secreted from the nerve terminal in order to initiate a number of downstream signalling pathways at the NMJ (14,15). Additionally, mutations in agrin itself can cause CMS, thus dysfunction in agrin action is already known to be pathological at the NMJ (13).

There are nine reported cases of agrin CMS to date, and only three for MYO9A, however, some phenotypic overlaps are present including the manifestation of both proximal and distal muscle weakness with respiratory involvement (6,13–15,51). Defining treatable-units is an important concept in the therapeutic intervention of rare diseases (such as CMS with NT-1654 as a promising drug). There are a number of features in MYO9A patients that do not appear in those with *AGRN* mutations, such as hypotonia, delayed motor milestones and ocular/bulbar involvement, therefore indicating that there are other factors involved in MYO9A pathophysiology as discussed above. Nevertheless, rescuing the agrin downregulation may lead to a valuable improvement to patient symptoms and has been shown to have beneficial effects in other disease models such as a severe SMA mouse in which a reduction in agrin had also recently been identified (52). A C-terminal, 44 kDa agrin fragment (NT-1654—Neurotune) engineered to be soluble, neurotrypsin resistant and still able to cluster AChRs, has been used with success to treat mice that have undergone disassembly of the NMJ in sarcopenia or nerve injury (16). Replacement of agrin through the expression of a motor neuron-specific transgene has also proven effective in mice with spinal muscular atrophy by increasing NMJ area and reducing intermediate filament accumulation (52). The SARCO mouse, a model of sarcopenia, also undergoes functional muscle strength improvements when treated with NT-1654 (16). Very recently Li and co-workers showed that NT-1654 attenuated clinical severity, effectively promoted the clustering of AChRs at NMJs, and alleviated the impairment of NMJ transmission, as well as the reduction of muscle-specific kinase (MuSK) in a rat model of experimental autoimmune myasthenia gravis (53). One of the most promising features of the NT-1654 treatment in particular is that it is exogenously applied, thus removing the requirement for complex gene therapy in patients. It also avoids the problem observed in the MYO9A-depleted cells of defects in vesicular transport and secretion, as it is clear that the cells are still synthesizing agrin due to the large intracellular accumulation of agrin present, but that it is not secreted by the nerve terminal. Thus, this fragment can act directly on the post-synaptic muscle fibre without initially relying on transport machinery in the nerve.

Mouse neural agrin displays a 54% homology with zebrafish, which increases to 60% when analysing only the Laminin G-like 2, EGF-like 4 and Laminin G-like 3 domains included within the NT-1654 compound. Replacement of agrin in the *myo9aa/ab* MO zebrafish using this mouse-derived fragment was successful in rescuing the movement defects we observe, as well as NMJ morphology. The improvement at 24 hpf of the chorion movements due to agrin injection implies it is rescuing the early development of the NMJ as expected, and we see further benefits at 48 hpf in speed and distance travelled when the zebrafish are able to swim away from a tactile stimulus. Acceleration of zebrafish is a direct measure of the force produced by a muscle contraction, and this is also improved, providing a functional measure that nerve to muscle transmission is working appropriately (19). Analysis of the NMJs of agrin-injected morphant fish revealed an improvement in neurite extension, which is impaired in fish lacking myo9aa/ab as previously reported (6). This suggests the addition of agrin is also improving the nerve extension, and corresponding pre-synaptic improvements are also reported in NT-1654-treated SARCO mice, as well as mice that have undergone a sciatic nerve crush injury (16). In a number of fish we also observe an increase in AChR staining, in both agrin only and agrin/MO zebrafish, which is unsurprising when considering the role of agrin in clustering of AChRs. While here we saw a high level of embryo death when co-injecting the MO and agrin, likely due to the amount of exogenous material being injected, it is important to note that this is just a proof of principal experiment demonstrating that agrin replacement ameliorates some of the NMJ phenotype we see *in vivo*. This supports our *in vitro* proteomic and cellular analysis data and provides a starting point for future development of therapeutic strategies that will be generated.

## Conclusions

In this study, our combined functional and biochemical studies utilizing NSC34-cells as a pre-synaptic *in vitro* system support the already speculated role of MYO9A in maintenance of the neuronal cytoskeleton and moreover demonstrated a detrimental downstream-effect on vesicular transport and protein secretion. As a result of this, we observe a lack of agrin secretion which could be rescued with application of an exogenous agrin fragment *in vivo*, thus leading to the identification of a protein of potential therapeutic impact for patients. Discovery of this underlying pathophysiology links two sub-types of CMS together (Agrin and MYO9A) with regards to their molecular basis. This connection highlights the need for in-depth molecular analysis of genes causing NMJ disorders to find common pathomechanisms for which we can invoke similar treatment strategies.

## Materials and Methods

### Cell culture

NSC-34 cells stably depleted for MYO9A using shRNA-mediated knockdown as described previously (6) and control knockdown cells were cultured to sub-confluence in Dulbecco’s modified Eagle medium (DMEM; Life Technologies) supplemented with 10% foetal bovine serum, 100 units/ml penicillin/streptomycin and 3 µg/ml puromycin to maintain selective pressure. To generate the control knockdown line, NSC-34 cells were transfected with a control shRNA (Santa Cruz Biotechnology, 108060) using Lipofectamine 2000 (ThermoFisher Scientific) according to manufacturer’s instructions, as previously described (6).

### Quantitative reverse transcriptase PCR

Control and MYO9A-depleted cells (5 × 10^6^) were trypsinized and homogenized using a Tissue Ruptor. Cells were transferred to an RNeasy Mini spin column (RNeasy Mini Kit, Quiagen) and RNA extracted according the manufacturer’s instructions. RNA was eluted in RNase-free water and quantified using a nanodrop. MYO9A depletion was confirmed by quantitative reverse transcription polymerase chain reaction (qRT-PCR) using *Power*SYBR Green Master Mix according to manufacturer’s instructions (Applied Biosystems, 4368706), as β-actin which had previously been used to normalize the qRT-PCR results has since been identified as dysregulated in the MYO9A-depleted cells. Here the qRT-PCR was repeated with another housekeeping gene; *PgK1* (5′-GGAGCGGGTCGTGATGA-3′ and 5′-GCCTTGATCCTTTGGTTGTTTG-3′) as has been performed elsewhere (24). Following confirmation of primer annealing temperature and reaction efficiency, three biological replicates in triplicate were run for *Myo9a* and *PgK1*, with a corresponding no template control. The relative expression ratio (RER) was determined using the formula:

$$RER\;of\;Myo9a=\frac{2^{-\Delta Cttarget(KD-control)}}{2^{-\Delta Ctref(KD-control)}}$$

where ΔCt is the difference in crossing points, ref is the corresponding value for the reference gene and KD refers to the MYO9A knockdown cells.

### Immunofluorescence in cells

NSC-34 cells (control and MYO9A-depleted) were fixed in 1:1 4% paraformaldehyde: growth medium at 37°C for 5 min and permeabilized in 0.1% Triton™ X-100 in phosphate buffered saline (PBS) for 10 min. Blocking was performed using 4% bovine serum albumin (BSA) in PBS with 0.1% Tween (PBS-T) at room temperature for 1 h. Anti-bodies used are shown in Supplementary Material, Table S1 and were diluted in 1% BSA in PBS-T, and washes carried out using PBS-T. Samples were mounted in ProLong Diamond Anti-fade Mountant with DAPI (Molecular probes). For cells treated with a ROCK inhibitor (InSolution™ Y-27632—Calbiochem), the compound was applied at 3 nm in serum-free growth medium over-night (cells without Y-27632 were also grown for 24 h in serum-free medium). Staining was then performed as outlined above. Cells were visualized using a Nikon A1R laser scanning confocal, with oil-immersion 40× or 60× objectives. In order to quantify the fluorescence intensity of various anti-bodies applied to NSC-34 cells, Fiji ImageJ was utilized to obtain average fluorescence, cell area and integrated density of individual cells at a 40× magnification. For each image, three regions without cells were also measured to establish a mean background fluorescence value. Corrected total cell fluorescence (CTCF) was then calculated using the equation below:

CTCF=Integrated density–(area of selected cell x mean fluorescence of background readings)

The presence or absence of stress fibre-like structures was manually assessed in cells imaged at 60× and 40× objectives. A directionality plugin on ImageJ was also utilized to determine the orientation of the actin filament network in the cells (http://imagej.net/Directionality; date last accessed November 5, 2017).

### Immunoblotting

Cells were washed three times in PBS before scraping cells directly into ice-cold lysis buffer [RIPA buffer with protease inhibitor tablet (Roche)] to generate whole protein extracts. Those were maintained at 4°C with constant agitation for 30 min, centrifuged for 20 min at 1750*g* at 4°C, then heated to 95°C for 5 min in 2× Laemmli buffer (Bio-Rad). Equal loading of samples was confirmed by Coomassie staining. Adjusted lysate quantities were then subjected to SDS-polyacrylamide gel electrophoresis (PAGE) and proteins transferred to polyvinylidene difluoride-membranes (Millipore). The membranes were blocked with 5% low-fat dried milk in Tris-buffered saline (TBS) and 0.1% Tween (TBS-T) for 1 h at room temperature. Primary anti-bodies were then applied over-night in blocking milk at 4°C, as listed in Supplementary Material, Table S1. Following TBS-T washes, membranes were incubated with secondary anti-bodies as shown in Supplementary Material, Table S1, for 1 h at room temperature. Following three washes in TBS-T and one wash in TBS, bound anti-bodies were detected using either the Odyssey CLx Imaging System (β-actin and β-tubulin), or enhanced chemiluminescent substrate (SuperSignal West Femto; Pierce, Neurofilament).

### Vesicular recycling assay

NSC-34 control and MYO9A-depleted cells were cultured to 80% confluency in six well plates on coverslips and transfected with 2 µg Tropomyosin receptor kinase A (TrkA)-GFP tagged constructs (kind gift from Dr Joachim Weis, Aachen) using lipofectamine 2000 reagent (ThermoFisher Scientific) according to manufacturer’s instructions. After 24 h incubation at 37°C, cells were washed twice with pre-warmed PBS and then serum-starved for 2 h. Growth medium containing 50 ng/ml of nerve growth factor (NGF) (Sigma–Aldrich) was then applied to stimulate internalization of TrkA receptors for 15 min. Serum- and NGF-free medium was then added to allow recycling of receptors back to the surface. Cells were fixed before NGF application (pre-NGF) and after NGF application at time points 0, 10, 30, 60 and 180 min in 1:1 4% paraformaldehyde: growth medium at 37°C for 5 min. Cells were mounted on slides with ProLong Diamond Anti-fade Mountant with DAPI (Molecular probes) and analysed using a Nikon A1R laser scanning confocal microscope. Cells were analysed at 50 per time point and the entire experiment repeated 4 times, giving a total of 200 cells per time point. In order to assess protein levels of TrkA throughout the experiment, cells were also seeded onto 10 cm^2^ plates and transfected with the TrkA constructs as above. Following application of NGF, cells were lysed at different time points as in the immunoblotting section and a western blot performed using the TrkA anti-body (Supplementary Material, Table S1). This was performed three times. Global endo and exocytosis was also assessed using FM 1–43, this is described in the Supplementary Material, Methods.

### Time-lapse imaging

NSC-34 control and MYO9A-depleted cells were seeded onto eight well glass bottom µ-slides (Ibidi) and transfected with the coding sequence of the *TrkA* receptor as in the recycling assay. After 24 h, a ROCK inhibitor (InSolution™ Y-27632—Calbiochem), was applied at 3nM in serum-free growth medium over-night and NucRed Live 647 ReadyProbes reagent (Invitrogen) was applied for visualization of nuclei. Cells were imaged using a Nikon A1R laser scanning confocal microscope over a period of ten minutes per cell, with z-stacks obtained. Resulting image series were then analysed using Imaris, with automatic detection of TrkA positive vesicles based on thresholding that was manually edited. Vesicles were then tracked by the software over-time and in 3D space to provide an output for speed the vesicles travelled, 10 cells were analysed per condition over three experimental repeats.

### Secretomic analysis of MYO9A-depleted cells

NSC-34 cells (three samples of both wild-type and MYO9A-depleted) were cultured to 80% confluency and washed three times with PBS. Serum-free growth medium was added for 4 h, followed by the addition of growth medium supplemented with 10% FBS for 1 h (pulse). Following three washes with PBS, serum-free medium was applied for 3 h (chase) and then collected and centrifuged for 4 min at 1750*g*. The supernatant was combined with 10× ice-cold ultrapure ethanol over-night at -80°C. Samples were then centrifuged at 18 000*g* for 30 min at 4°C and the pellets dried under laminar hood flow before the addition of SDS lysis buffer (50 mm Tris–HCl pH 7.8, 150 mm NaCl, 1% SDS, 1 protease cOmplete mini tablet and one phosphatase inhibitor PhosSTOP tablet, Roche). Samples were subjected to quantitative mass spectrometry as outlined in Supplementary Material, Methods. Criteria for defining a protein as regulated are: protein commonly quantified in each replicate, identified by at least one unique peptide, an ANOVA *P*-value of <0.05 and an average log 2 ratio of either lower than the down-regulated cut-off or higher than the up-regulated cut-off (<-4.44 or >4.06).

### Zebrafish morpholino injections and NT-1654 treatment

Morpholino-mediated knockdown of zebrafish embryos was performed with an anti-sense morpholino oligonucleotide (MO) as previously described for the zebrafish *MYO9A* orthologues; *myo9aa* and *myo9ab* (6). Briefly, knockdown was performed on one-cell stage embryos of the AB strain of zebrafish and they were either uninjected, injected with NT-1654 alone, MO alone or NT-1654 and MO. The artificial agrin compound, NT-1654, is a 44 kDa fragment of neural agrin that is soluble and resistant to neurotrypsin cleavage, developed by Neurotune (16). Embryos from the same pair of fish were split into each of the four categories to ensure fair comparisons for survival and developmental rate. At 24 h post-fertilization (hpf), zebrafish from each condition were recorded for 1 min per dish (>40 zebrafish per condition) and movements within the chorion recorded per fish. This was performed using a Leica stereomicroscope with a Chameleon digital camera (CMLN-13s2M). At 48 hpf zebrafish were again recorded following dechorionation and a period of acclimatization, to quantify their response to tactile stimulation of the back of the head using a fine pipette tip (average 34 zebrafish per condition), as described (19). Videos were obtained using a Canon legria hfr76 camera at 25 frames per second. Embryos were placed individually in a petri dish containing E3 medium (5 mm NaCl, 0.17 mm KCl, 0.33 mm CaCl_2_, 0.33 mm MgSO_4_ and 10 μg/100 ml Methylene Blue) on an illuminated stage. Room temperature remained constant at 28°C throughout the experiment. The video camera was held using a clamp at 7 cm above the dish for every experiment and a ruler was used to adjust the scale used for measuring in ImageJ for each repeat. Fiji ImageJ was utilized to quantify the data obtained, with files imported into ImageJ as FFmpeg movies and thresholding applied to allow visualization of the zebrafish followed by conversion to a binary image. The Trackmate plugin was then utilized to measure the movement of the zebrafish, with manual editing of each reading performed to ensure the zebrafish is tracked correctly through each video frame. Values for average speed, acceleration and total distance travelled were obtained and compared between treatment groups.

### Immunofluorescent staining of whole mount zebrafish

At 48 hpf, zebrafish embryos from each treatment group were dechorionated using Pronase E (Sigma–Aldrich) and euthanized by anaesthetic over-dose. Whole mount staining was performed as described previously (54), utilizing a mouse anti-SV2 anti-body to visualize the motor neurons (1:200, Developmental Studies Hybridoma Bank) and Alexa Fluor 488-α-bungarotoxin conjugate (1:1000, Life technologies). Z-stack images encompassing the entire zebrafish tail were obtained using a 20× air objective on a Nikon A1R confocal microscope.

### Statistics

Statistical analysis was performed using GraphPad Prism software and either the Mann–Whitney or unpaired *t*-test was utilized, following assessment for normal distribution. Statistical significance was taken as *P* < 0.05. *In vitro* experiments were blinded for image analysis and *in vivo* experiments blinded prior to live recording and for image acquisition.

## Ethical Approval

All applicable international, national and institutional guidelines for the care and use of animals were followed. This article does not contain any studies with human participants performed by any of the authors.

## Supplementary Material

Supplementary Material is available at *HMG* online.

## Supplementary Material

Supplementary Data
