# Supplementary material for: MYO9A deficiency in motor neurons is associated with reduced neuromuscular agrin secretion
Source: Hum Mol Genet. 2018 Feb 16;27(8):1434–46. doi: 10.1093/hmg/ddy054 (PMC5991207; doi:10.1093/hmg/ddy054)
Supplement: Supplementary Data [file ddy054_supplementary_material.docx]

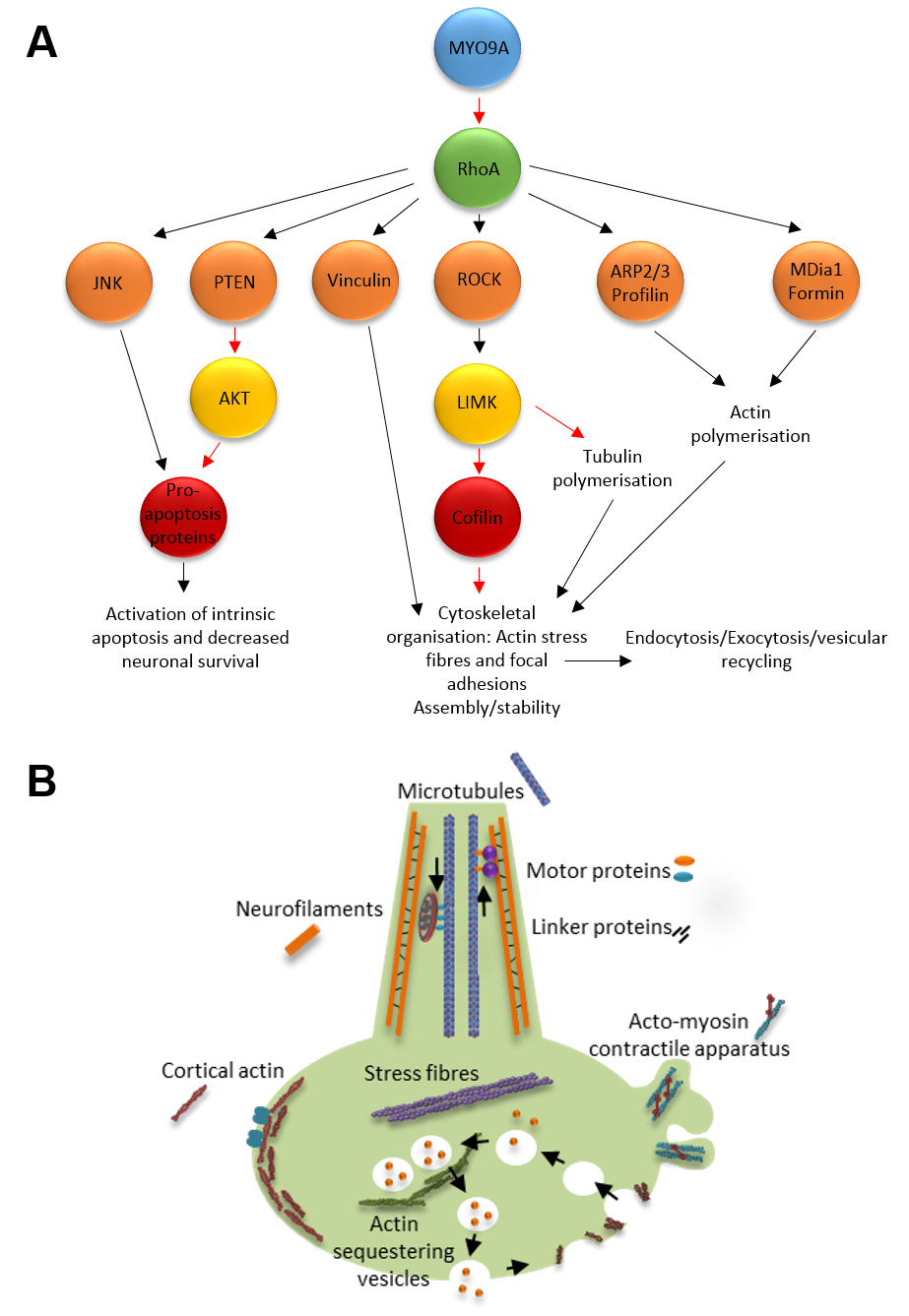


**Supplementary Figure 1: Influence of MYO9A on the RhoA signalling pathway and outline of nerve cell cytoskeleton.** MYO9A can act as a negative regulator of RhoA and thus influence numerous downstream pathways. Here some of the signalling mechanisms involved in apoptosis and cytoskeletal dynamics are shown, however, RhoA pathways are extensive. JIP3 is shown due to its downregulation in proteomic data for MYO9A KD cells. Red arrows = negative regulation, black arrows = positive regulation. **B:** Schematic of nerve cell cytoskeleton, including microtubules for long distance axonal transport and neurofilament for structural stability. Various confirmations of actin are also shown, such as cortical actin involved in shape regulation and signalling, stress fibres, acto-myosin contractile apparatus and actin fibres for sequestration of vesicle reserve pools.

| Antibody type | Antibody | Source | Concentration |
| --- | --- | --- | --- |
| Primary | Mouse monoclonal anti-neurofilament | ThermoFisher Scientific | 1:100 |
| Primary | Rabbit polyclonal anti-PRX | Bioss antibodies | 1:100 |
| Primary | Rabbit polyclonal anti-β-tubulin | Abcam | 1:100 |
| Primary | Mouse monoclonal anti-β-actin | Sigma | 1:1000 |
| Primary | Mouse monoclonal anti-agrin | Santa Cruz | 1:100 |
| Direct | Oregon Green 488 phalloidin | Life Technologies | 1:1000 |
| Secondary | Alexa Fluor® 488 goat anti-rabbit | Life Technologies | 1:500 |
| Secondary | Alexa Fluor® 594 goat anti-rabbit | Life Technologies | 1:500 |
| Secondary | Alexa Fluor® 594 goat anti-mouse | Life Technologies | 1:500 |
| Secondary | DyLight 680 goat anti-mouse | ThermoFisher | 1:2000 |
| Secondary | DyLight 680 goat anti-rabbit | ThermoFisher | 1:2000 |
| Secondary | Goat anti-mouse HRP conjugated | Invitrogen | 1:2000 |

**Supplementary Table 1:** List of antibodies used in experiments described, including source and concentration utilised.

**
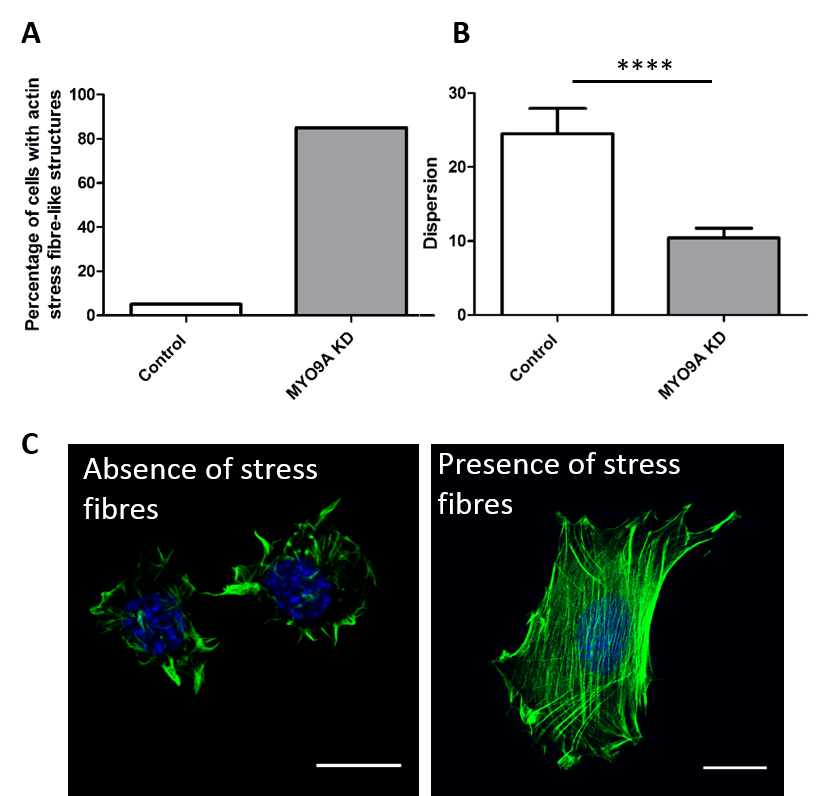
**

**Supplementary Figure 2: Assessment of actin stress fibre-like structures in cells depleted for MYO9A. A:** Percentage of control and MYO9A-depleted NSC-34 cells that have actin stress fibre-like structures present (n>65). **B:** Orientation of actin positive fibres assessed using the ImageJ Directionality plugin. The dispersion of the fibre direction is shown (low dispersion = increased organisation). Error bars represent mean + standard error of the mean, unpaired t-test, ****p<0.0001 (n>65). **C:** Example images showing absence and presence of actin stress fibre-like structures. Scale bars = 20µm.

## FM 1-43 Dye experiment

NSC-34 control and MYO9A-depleted cells were cultured so sub-confluence before seeding 0.1 x 10^6^ cells into 12 well plates with coverslips and left overnight to adhere. FM 1-43 working stock (5μg/mL) was made up in ice cold Hank's Balanced Salt Solution (HBSS) without magnesium or calcium. Dye added to wells of plate sequentially to give staining times of 1-18 minutes, with the plate incubated at 37°C between addition of staining solution. After 18 minutes, cells were washed with ice cold HBSS and fixed in 4% paraformaldehyde in HBSS for 10 minutes on ice. Coverslips were removed from wells and applied to slides with mounting medium containing DAPI (Vectashield) before imaging using a Nikon AR1 Confocal. Z-stacks throughout the cells were obtained, with 4 separate fields of view imaged at each time point for each cell type and subsequently quantified for fluorescence using ImageJ (over 36 cells per condition). Corrected total cell fluorescence was used, as outlined in the main paper.

## Proteomics

### Sample preparation and tryptic digestion

Three MYO9A-depleted NSC34 and three NSC34 control secretome samples were used for the comparative proteome profile, using the medium from T75 tissue culture flasks. The secretome was incubated with 10 x ethanol (v/v) for 4 hours at -20 °C, and subsequently centrifuged at 18.000 xg, 4 °C for 30 minutes. Proteins were then resuspended and lysed in 300 µL of 50 mM Tris-HCl (pH 7.8) buffer containing 150 mM NaCl, 1 % SDS, supplemented with complete mini EDTA-free and phosphoStop (Roche). Following a centrifugation step at 13,500 x g and 4 °C for 30 min, supernatant was collected and protein concentration was determined by BCA assay according to the manufacturer’s protocol (Pierce BCA Protein Assay Kit, Thermo Scientific). Afterwards, protein cysteines were reduced with dithiothreitol (F.c 10 mM DTT) at 56 °C for 30 minutes, followed by alkylation of new generated free thiols with iodoacetamide (F.c. 30 mM IAA) at room temperature (RT) in the dark for 30 min.

Sample preparation and proteolysis were performed using filter-aided sample preparation (FASP). Briefly, 100 µg of protein was diluted 10-fold with freshly prepared 8 M urea/100 mM Tris-HCl (pH 8.5) buffer and placed onto a 30 kDa weight cut-off (MWCO) centrifugal device (Pall Corporation, Nanosep). The device was centrifuged at 13,500 g at RT for 20 min for all of the centrifugation steps. First, three washing steps were carried out to eliminate residual SDS. Afterwards, three more centrifugation steps were performed with 100 µL of 50 mM aminobicarbonate (ABC, NH_4_HCO_3_) (pH 7.8) to exchange the buffer. Lastly, 100 µL of proteolysis buffer comprising of 4 µg trypsin (Promega, Trypsin Gold, Mass Spectrometry Grade) in 0.2 M GuHCl, 2 mM CaCl_2_ and 50 mM ABC (pH 7.8), was added to the device and incubated at 37°C for 14 h. Following the collection of newly generated tryptic peptides by centrifugation with 50 µL of 50 mM ABC and 50 µL of ultra-pure water, the peptides were acidified to completely inactivate trypsin by adjusting the pH to less than 3 with 10 % TFA (v/v). Prior subjection to MS analysis, the digestion efficiency was controlled by reversed-phased Ultimate 3000 RSLC 200 µm x 5 cm.

### LC-MS/MS and label-free analysis of secretome Myo9A-depleted NSC34

The secretome replicates were measured using an Ultimate 3000 nano RSLC system with Acclaim PepMap 100 µm x 2 cm, C18, 5 µm, 100 Å traping column for 10 min using 0.1% TFA (v/v), flow rate of 20 µl/min, followed by a separation on Acclaim PepMap RSLC 75 µm x 50 cm, C18, 2 µm, 100 Å main column coupled to an Orbitrap Elite Mass Spectrometer (all from Thermo Scientific) with a 115 min LC gradient ranging from 3 to 45% B (84% ACN in 0.1% FA) at a flow rate of 250 nL/min. MS survey scans were acquired in the Orbitrap from m/z 300 to 1500 at a resolution of 60,000 using the polysiloxane ion at m/z 371.101236 as lock mass. The ten most intense signals were subjected to collision induced dissociation (CID) in the ion trap, taking into account a dynamic exclusion of 30 s. CID spectra were acquired with a normalized collision energy of 35 % and an activation time of 10 ms. AGC target values were set to 10^6^ for Orbitrap MS and 10^4^ for ion trap MSn scans, and maximum injection times were set to 100 ms.

Data analysis of the acquired label free quantitative MS data was performed using the Progenesis Qi software from Nonlinear Dynamics (Newcastle upon Tyne, U.K.) in which alignment of MS raw data was conducted by automatically selecting one of the LC-MS files as reference. After peak picking, only features within retention time and m/z windows from 0-115 min and 300-1500 m/z, with charge states +2, +3, and +4 were considered for peptide statistics and analysis of variance (ANOVA). MS/MS spectra were exported in an mgf file as peak lists.

MS/MS spectra were exported in an mgf file and searched against a concatenated target/decoy version of the mouse Uniprot database, (downloaded on 22/07/15, containing 16,473 target sequences) using Mascot 2.4.0 (Matrix Science), X! TANDEM Vengeance (15/12/15) and MS-GF+ Beta (v10282) (19/12/2014) with the help of searchGUI 3.2.5. Tryptic peptides with a maximum of two missed cleavages were chosen. Carbamidomethylation of cysteine was set as fixed and oxidation of methionine was selected as a variable modification. MS and MS/MS tolerances were 10 ppm and 0.5 Da, respectively.

The PeptideShaker software 1.4.0 (<http://code.google.com/p/peptide-shaker/>) was used for interpretation of peptide and protein identifications. Combined search results were filtered at a false discovery rate (FDR) of 1 % on the protein level and exported and re-imported into Progenesis Qi. Oxidized methionine peptides were excluded and only proteins that were quantified with unique peptides were exported. Then, for each protein, the average of the normalized abundances (obtained from Progenesis Qi) from the replicate analyses was calculated to determine the ratios between the Myo9A-depleted NSC34 samples and the controls. Only proteins which were (i) commonly quantified in all the replicates with (ii) at least one unique peptides, (iii) an ANOVA p-value of <0.05 (Progenesis Qi) and (iv) an average log 2 ratio of which protein that was either higher than the up-regulated cut-off or lower than the down-regulated cut-off was considered as regulated. The cut-off values were determined based on the 2x standard deviation and the normal distribution from all identified protein’s log2 ratio in which the bell curve is symmetric around the mean. Therefore, an average log2 ratio of a protein < -4.44 or > 4.06 (corresponding to ~16.7-fold regulation; log2 ratios of -0.19) for comparative secretome profile were considered as regulated.


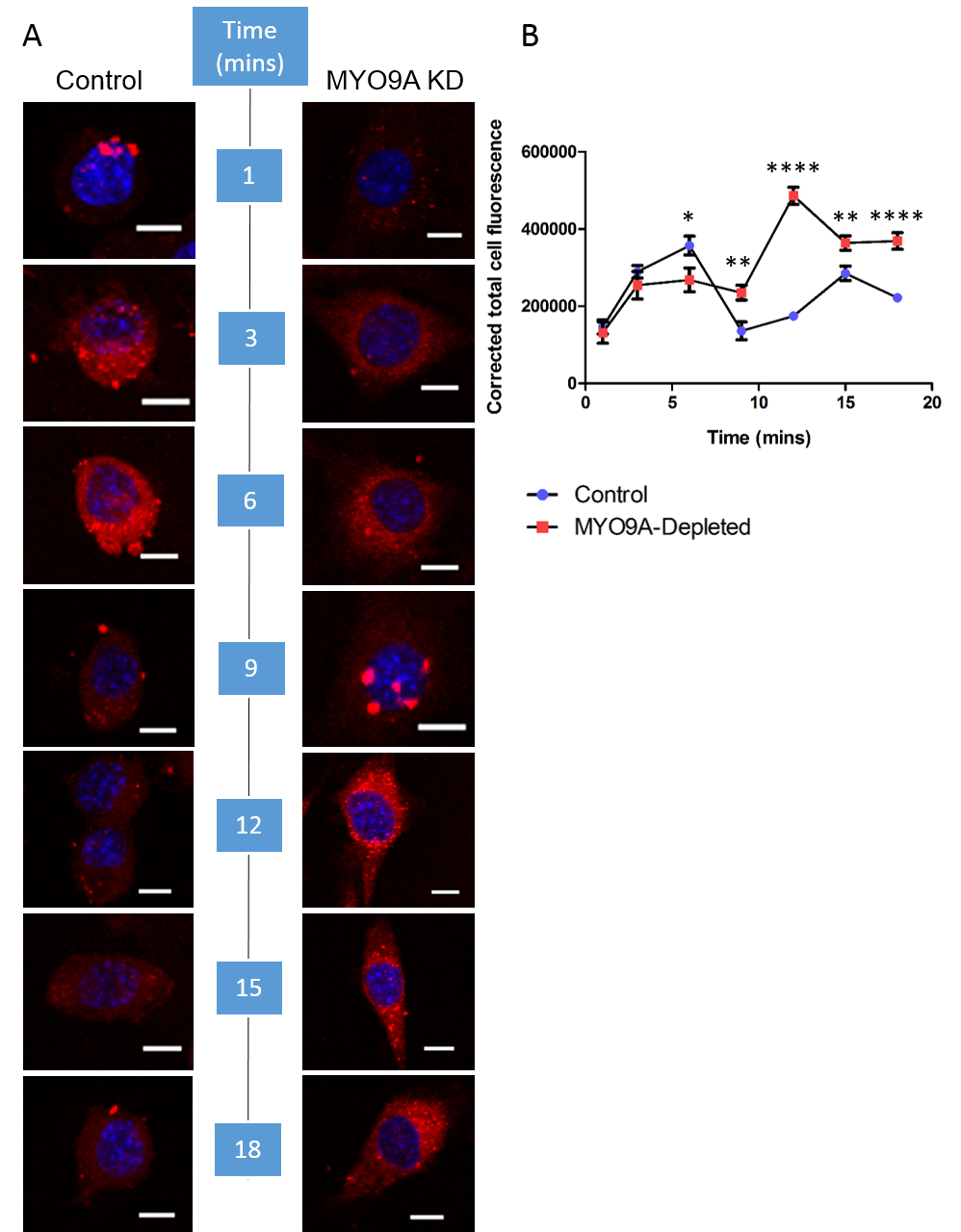


**Supplementary Figure 3: Global endocytosis and exocytosis is dysregulation in MYO9A-depleted cells. A:** FM 1-43 dye experiment in which cells were exposed to the dye for 1 to 18 minutes before fixation for quantification at different time points. Increased uptake of dye can be observed in control cells in the first 6 minutes, with uptake slower in the MYO9A KD cells, that peaks around 12 minutes. Scale bar = 10µm. **B:** Graph displaying the corrected total cell fluorescence at different time points, Unpaired t-test, *p≤0.05, **p≤0.01, ***p≤0.001, ****p<0.0001, error bars = ± standard error of the mean, n>36.

## Proteins identified in secretomic experiment

| Protein | Accession number | Localisation | Function | Disease involvement | Fold of regulation |
| --- | --- | --- | --- | --- | --- |
| MIME | Q62000 | secreted | Bone formation and axonogensis |  | 8.84108552 |
| PPIC | P30412 | secreted | Protein folding |  | 8.129945779 |
| HMOX1 | P14901 | secreted | Cytoprotection | Heme oxygenase 1 deficiency | 6.962838522 |
| LPP | Q8BFW7 | secreted | Cell shape and motility |  | 6.490882985 |
| MYOF | Q69ZN7 | secreted | Plasmalemma repair and endocytic recycling |  | 6.405636168 |
| PDLI1 | O70400 | cytoskeleton | Cytoskeleton |  | 6.373342417 |
| GAS1 | Q01721 | cell membrane | Cell growth suppressor |  | 6.178146105 |
| SODE | O09164 | secreted | Destroys free radicals | Amyotrophic Lateral Sclerosis 1 | 6.099830689 |
| CO4A1 | P02463 | secreted | Interact with laminins, proteoglycans and entactin/nidogen | Brain small vessel disease with or without ocular anomalies , Hereditary angiopathy with nephropathy aneurysms and muscle cramps, Porencephaly 1, Intracerebral hemorrhage, Schizencephaly | 6.046721552 |
| MMP2 | P33434 | secreted | Angiogenesis and tissue repair | Multicentric osteolysis, nodulosis, and arthropathy | 5.849368216 |
| SRPX2 | Q8R054 | secreted | Promotes synapse formation | Rolandic epilepsy with speech dyspraxia and mental retardation X-linked | 5.70959609 |
| SH3K1 | Q8R550 | secreted | Endocytosis, lysosomal degradation, cellular stress responses, apoptosis and cytoskeletal organization |  | 5.698131221 |
| NDRG1 | Q62433 | secreted | Cell growth, differentiation, apoptosis, cell trafficking, myelin sheath, vesicular recycling and regulating microtubule dynamics | Charcot Marie Tooth disease type 4D | 5.678838973 |
| CAPG | P24452 | secreted | Blocks the barbed ends of actin filaments |  | 5.677222105 |
| CO6A1 | Q04857 | secreted | Cell-binding protein. | Bethlem myopathy 1 and Ullrich congenital muscular dystrophy 1 | 5.587756594 |
| ENOB | P21550 | secreted | Striated muscle development and regeneration | Glycogen storage disease 13 | 5.556672165 |
| ANGP1 | O08538 | secreted | Angiogenesis, endothelial cell survival, proliferation, migration, reorganization of the actin cytoskeleton |  | 5.543351694 |
| S10A4 | P07091 | secreted |  |  | 5.524298487 |
| PRELP | Q9JK53 | secreted | Anchor basement membrane to connective tissue |  | 5.508115793 |
| OSTP | P10923 | secreted | Part of bone mineralized matrix |  | 5.366001405 |
| IBP6 | P47880 | secreted | Negative regulation of wnt WNT and cell proliferation |  | 5.353125727 |
| SC65 | Q8K2B0 |  |  |  | 5.34165765 |
| EMIL2 | Q8K482 | secreted | Anchoring smooth muscle cells to elastic fibers |  | 5.327777219 |
| CO3A1 | P08121 | secreted | Cortical development, inhibits neuronal migration and activates the RhoA pathway |  | 5.198026478 |
| PLTP | P55065 | secreted | Transfer of lipid molecules |  | 5.181661752 |
| LAMA4 | P97927 | secreted | Attachment, migration and organization of cells into tissues during embryonic development |  | 5.148926308 |
| VIME | P20152 | secreted | Intermediate filament, regulates microtubule dynamics | Cataract 30 | 4.949931945 |
| SPB6 | Q60854 | secreted | Regulation of serine proteinases | Deafness, autosomal recessive, 91 | 4.938047641 |
| RL22L | Q9D7S7 | secreted | Structural constituent of ribosome |  | 4.726654112 |
| S10AA | P08207 | secreted | Positive regulation of stress fiber assembly |  | 4.673247388 |
| CO5A2 | Q3U962 | secreted | Connective tissue component | Ehlers-Danlos syndrome, classic type | 4.661757006 |
| ANXA3 | O35639 | secreted | Inhibitor of phospholipase A2 |  | 4.62943553 |
| GSTA4 | P24472 |  | Conjugation of reduced glutathione to electrophiles |  | 4.585769672 |
| CO1A2 | Q01149 | secreted | Rho protein signal transduction | Ehlers-Danlos syndrome 7B, Osteogenesis imperfecta 1 and Osteogenesis imperfecta 2 | 4.572424063 |
| CADH2 | P15116 | secreted | Calcium-dependent cell adhesion proteins |  | 4.51399316 |
| HTRA1 | Q9R118 | secreted | Serine protease | Macular degeneration, age-related, 7, Cerebral arteriopathy, autosomal recessive, with subcortical infarcts and leukoencephalopathy | 4.483872692 |
| AEBP1 | Q640N1 | secreted | Enhanced adipocyte proliferation, reduced differentiation, binds calmodulin and is involved in protein processing |  | 4.47579898 |
| PEDF | P97298 | secreted | Neurotrophic protein; induces extensive neuronal differentiation, negative regulator of neuronal death | Osteogenesis imperfecta 6 | 4.423969538 |
| LTBP2 | O08999 | secreted | Structural role in elastic-fiber organization | Glaucoma 3, primary congenital, D, Microspherophakia and/or megalocornea, with ectopia lentis and with or without secondary glaucoma and Weill-Marchesani syndrome 3 | 4.33299379 |
| LYOX | P28301 | secreted | Oxidative deamination of peptidyl lysine residues in precursors to fibrous collagen and elastin | Aortic aneurysm, familial thoracic 10 | 4.291993841 |
| INF2 | Q0GNC1 | cytoplasm | Severs actin filaments and accelerates their polymerization and depolymerization | Focal segmental glomerulosclerosis 5, Charcot-Marie-Tooth disease, dominant, intermediate type, E | 4.273975547 |
| LAMB1 | P02469 | secreted | Organization of cells into tissues during embryonic development and architecture of cerebral cortex. | Lissencephaly 5 | 4.191424881 |
| NID1 | P10493 | secreted | Cell to extracellular matrix interactions |  | 4.186691014 |
| NQO1 | Q64669 | secreted | Negative regulation of apoptotis |  | 4.186185145 |
| FKB10 | Q61576 |  | Accelerate protein folding | Osteogenesis imperfecta 11, Bruck syndrome 1 | 4.111759089 |
| GMDS | Q8K0C9 | secreted | Catalyzes the conversion of GDP-D-mannose to GDP-4-dehydro-6-deoxy-D-mannose |  | 4.079698454 |
| IGEB | P03975 | cytoplasm |  |  | -4.44442495 |
| DOPO | Q64237 | secreted | Converts dopamine to noradrenaline | Cardiac Regulation | -4.449643808 |
| IGSF8 | Q8R366 | secreted | Mayb regulate neurite outgrowth in the adult brain |  | -4.518081271 |
| MRP | P28667 | secreted | Regulates actin cytoskeleton and neuronal migration |  | -4.520508705 |
| LAT1 | Q9Z127 | secreted | Involved in cellular amino acid uptake and neurogensis in the brain |  | -4.533051028 |
| UCHL1 | Q9R0P9 | secreted | Processing of ubiquitin precursors and of ubiquitinated proteins | Alzheimers Disease | -4.538729128 |
| BGLR | P12265 | secreted | Degradation of dermatan and keratan sulfates |  | -4.542954696 |
| COTL1 | Q9CQI6 | secreted | Binds to F-actin in a calcium-independent manner | Parkinsons Disease | -4.549514023 |
| H4 | P62806 | nucleus | Core component of nucleosome |  | -4.549850244 |
| DPYL5 | Q9EQF6 | cytoplasm | Neuronal differentiation and axon growth |  | -4.586679792 |
| SCG2 | Q03517 | secreted | Secretory granule protein | Parkinsons Disease | -4.768512141 |
| SCG3 | P47867 | secreted | Secretory granule protein | Parkinsons Disease | -5.120730002 |
| VAT1L | Q80TB8 | cytoplasm | Secretory granule protein | Parkinsons Disease | -5.205433024 |
| AKA12 | Q9WTQ5 | cytoplasm |  |  | -5.215792684 |
| AGRIN | A2ASQ1 | secreted | Formation and the maintenance of the neuromuscular junction | Congenital Myasthenic Syndrome | -5.357776055 |
| GNA13 | P27601 | secreted | Transmembrane signalling |  | -5.395246419 |
| TICN2 | Q9ER58 | secreted | May participate in neurogenesis |  | -5.636804699 |
| APLP1 | Q03157 | secreted | Postsynaptic function and regulation of neurite outgrowth |  | -5.790748164 |
| H2AY | Q9QZQ8 | secreted | Represses transcription |  | -5.833594691 |
| SCG1 | P16014 | secreted | Mediates the subcellular compartmentation of protein kinase A and C |  | -5.861864664 |
| MUC18 | Q8R2Y2 | secreted | Cell adhesion |  | -5.952142061 |
| CLUS | Q06890 | secreted | Prevents aggregation of nonnative proteins |  | -5.993152807 |
| PCSK1 | Q9QXV0 | secreted | Neuroendocrine secretory pathway |  | -6.222680148 |
| ELN | P54320 | secreted | Structural protein |  | -6.64445921 |
| DKK1 | O54908 | secreted | Antagonizes Wnt signaling |  | -7.281956859 |
| AAAT | P51912 | secreted | Amino acid transporter |  | -7.381076859 |
| LDHB | P16125 | secreted | Involved in synthesis of (S)-lactate from pyruvate. |  | -7.627742699 |
| EF1A2 | P62631 | nucleus | Promotes binding of aminoacyl-tRNA to ribosomes during protein synthesis |  | -7.628200738 |
| ITA6 | Q61739 | secreted | Receptor for laminin on platelets |  | -7.644407161 |
| CGL | Q8VCN5 | secreted | Catalyses the last step in the trans-sulfuration pathway |  | -7.926937788 |

**Supplementary Table 2. Proteins identified in secretomic experiment comparing proteins secreted from MYO9A-depleted NSC-34 cells with control NSC-34 cells.** The affected protein is listed along with the corresponding accession number, whether the protein has been reported as secreted (Uniprot, Pubmed, LOCATE and MGI) or otherwise the main cellular compartment where it resides, brief functional and disease involvement information obtained from UniProt, and the Log2 value showing the degree of dysregulation.
